# Extrachromosomal DNA (ecDNA) in cancer: mechanisms, functions, and clinical implications

**DOI:** 10.3389/fonc.2023.1194405

**Published:** 2023-06-28

**Authors:** Yucheng Dong, Qi He, Xinyu Chen, Fan Yang, Li He, Yongchang Zheng

**Affiliations:** ^1^ Chinese Academy of Medical Sciences & Peking Union Medical College, Beijing, China; ^2^ Department of Liver Surgery, State Key Laboratory of Complex Severe and Rare Diseases, Peking Union Medical College Hospital, Chinese Academy of Medical Sciences and Peking Union Medical College, Beijing, China; ^3^ Department of Pathology, University of Oklahoma Health Sciences Center, Oklahoma, OK, United States

**Keywords:** ecDNA, liquid bioposy, drug resistance, tumor evolution, NGS

## Abstract

Extrachromosomal DNA (ecDNA) is circular DNA that plays an important role in the development and heterogeneity of cancer. The rapid evolution of methods to detect ecDNA, including microscopic and sequencing approaches, has greatly enhanced our knowledge of the role of ecDNA in cancer development and evolution. Here, we review the molecular characteristics, functions, mechanisms of formation, and detection methods of ecDNA, with a focus on the potential clinical implications of ecDNA in cancer. Specifically, we consider the role of ecDNA in acquired drug resistance, as a diagnostic and prognostic biomarker, and as a therapeutic target in the context of cancer. As the pathological and clinical significance of ecDNA continues to be explored, it is anticipated that ecDNA will have broad applications in the diagnosis, prognosis, and treatment of patients with cancer.

## 1 Introduction

Cancer cell evolution and tumour heterogeneity are among the greatest challenges in cancer treatment, fueling both innate and adaptive responses to anti-cancer drugs. Gain-of-function mutations of oncogenes and drug resistance genes, as well as downregulation of tumour suppressor genes, are among the genetic changes that contribute to this adaptability. In addition, large-scale changes in the genome such as amplifications, deletions, and loss of heterozygosity are frequently observed in tumors. It is also now well documented that dysregulation at the epigenetic, transcriptional, translational, and post-translational levels can substantially affect cancer cell survival and tumor progression.

Although its significance is rapidly gaining recognition, the role of extrachromosomal DNA (ecDNA)—circular DNA constructed from nucleosomal chromatin—in cancer is less well characterized. ecDNA has been identified as a major carrier of amplified oncogenes (1), and it is more open than chromosomal DNA, resulting in increased transcriptional activity (2). Owing to its lack of a centromere, ecDNA is inherited in a non-Mendelian pattern during mitosis (3), resulting in some tumour cells carrying high copy numbers of oncogenes that can substantially influence tumor heterogeneity and drug resistance (4). In recent studies using whole-genome sequencing (WGS) and cytogenetics approaches, ecDNA was found in various types of cancer, but rarely in normal tissues (5). Furthermore, studies have documented that ecDNA can be associated with more aggressive tumours and worse clinical prognosis (6). Thus, as the mechanisms of ecDNA dynamics and their role in cancer are gradually being elucidated, it is highly anticipated that drugs targeting ecDNA formation and maintenance may be useful as cancer therapeutics.

Previous reviews explained the roles of ecDNA in cancer progression and regulation of ecDNA (7–10), or focused on the functions of ecDNA in specific types of cancer, such as colorectal cancer (11) and glioblastoma (12). In this review, we discuss the molecular features, functions, mechanisms of formation, and detection methods of ecDNA, with a focus on the potential clinical implications of ecDNA in cancer. Specifically, we consider the role of ecDNA in acquired drug resistance, as a diagnostic and prognostic biomarker, and as a therapeutic target in the context of cancer. As the pathological and clinical significance of ecDNA continue to be explored, it is anticipated that ecDNA will have broad applications in the diagnosis, prognosis, and treatment of patients with cancer.

## 2 Molecular features, formation, and functions of ecDNA

### 2.1 Structure of ecDNA

In the 1980s, several transmission electron microscopy (TEM) studies suggested that ecDNA was circular in shape and consisted of chromosomal fibers containing nucleosomes (13–15). In 2019, Wu et al. uncovered additional details regarding the structure of ecDNA by combining ultrasound imaging, optical imaging, and WGS analysis (16), ultimately determining that ecDNA tended to be less compact and more accessible than chromosomal DNA, resulting in increased expression of ecDNA loci (16). In addition, this study suggested that ecDNA could be present in high copy numbers, leading to significant overexpression of genes, including oncogenes (16). Similarly, a WGS analysis performed by Kim et al. showed that amplification of ecDNA molecules resulted in higher expression of oncogenes compared with linear DNA (6). Therefore, ecDNA is currently characterized as circular molecules with relatively de-compacted chromatin, leading to increased levels of gene expression.

### 2.2 Formation of ecDNA

**Figure 1:**
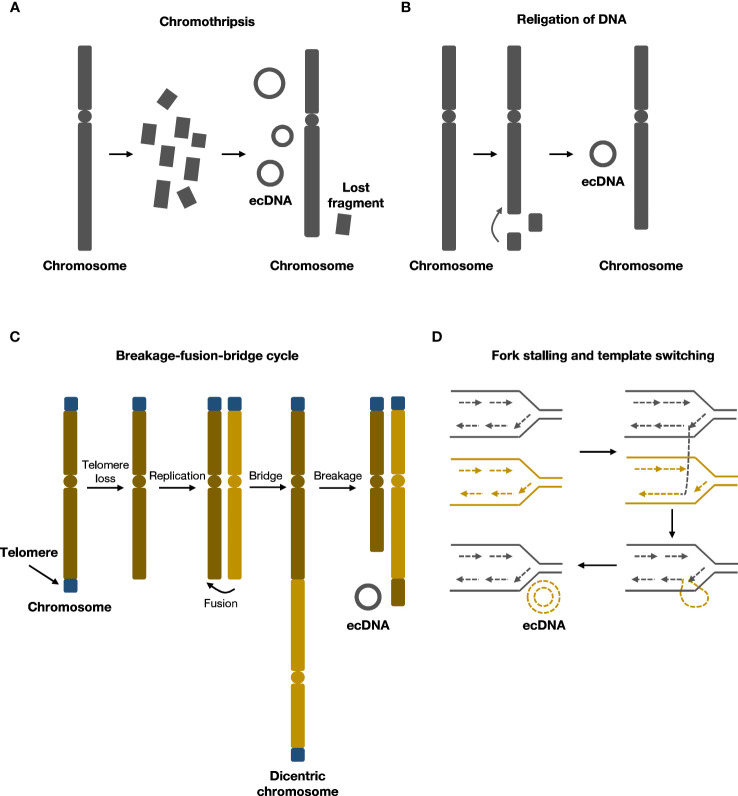
Formation mechanisms of ecDNA. **(A)** During chromothripsis, chromosomes are shattered and religated to form ecDNA.**(B)** Local religation of DNA can form ecDNA. **(C)** Breakage-fusion-bridge (BFB) cycles generate rearranged chromosomes and ecDNA. **(D)** Fork stalling and template switching can loop out to form ecDNA.

Previous studies have indicated a strong correlation between ecDNA formation and genome instability (17, 18). Recent research has shown that various molecular events associated with chromosomal damage can lead to the formation of ecDNA. These events include chromothripsis, religation of DNA, breakage-fusion-bridge (BFB) cycles, fork stalling, and template switching (**Figure 1**).

#### 2.2.1 Chromothripsis

Chromothripsis refers to chromosome shattering events wherein tens to hundreds of locally clustered DNA breaks occur in one or a few chromosomes simultaneously, resulting in DNA fragment rearrangements and widespread loss of DNA sequences (19). During this process, the rearrangement of chromosomes can cause small circular DNA fragments to form, including ecDNA, also known as ‘double minutes’ (19–21). Chromothripsis is a singular, one-off event that differs from multiple successive DNA rearrangements in punctuated equilibrium events such as the breakage–fusion–bridge cycle (19). Recent evidence supporting this chromothripsis model of ecDNA formation includes the finding that, in human lymphoma cell lines, 6.3% of the circular junction sequences within ecDNA contained microhomologies, suggesting that microhomology-mediated DNA repair occurred during the generation of circular DNA (22). Similarly, in neuroblastoma cell lines, 2.8% of circular junction sequences contained nontemplate insertions, indicating nonhomologous end-joining (NHEJ) repair or replication-associated mechanisms were involved in ecDNA formation (23).

ecDNA generated by chromothripsis may harbor oncogenes that could confer a proliferative advantage (19). Moreover, in addition to the random, non-Mendelian inheritance of ecDNA during cell division, ecDNA can be further amplified within the host cell (19). Various environmental pressures, such as anti-cancer drugs and immune surveillance, usually result in cancer cells eventually containing high ecDNA copy numbers (24). Previous studies have also shown that enhancer elements can be integrated into ecDNA during their generation, and these sequences can play important roles in oncogene expression (25). Further, it has been demonstrated that ecDNA is characterized by a higher mutation rate during replication as well as lower repair efficiency than genomic DNA, and that repair of DNA damage on ecDNA is likely to result in point mutations and small indels (26).

#### 2.2.2 Religation of DNA

Although chromothripsis is considered the main mechanism of ecDNA formation, chromothripsis signatures are present in only about 36% of the precursor DNA segments that form ecDNA (6), suggesting that there are other mechanisms of ecDNA formation.

In human cell lines, researchers were able to generate ecDNA from DNA fragments deleted from chromosomes using CRISPR–Cas9 system (27). In addition, a case study showed that amplification of *MYC* on ecDNA coincided with deletion of *MYC* on chromosomes from patients with leukaemia, suggesting that ecDNA may originate from chromosomal DNA deletions (28). Similarly, in glioma cells, sequence analysis revealed that DNA fragments from chromosomal deletions or rearrangements could form ecDNA by a mechanism similar to V(D)J recombination (29). Therefore, one model for another mechanism of ecDNA formation proposes that deleted chromosomal DNA could ligate to form ecDNA without chromothripsis, but further research is needed to delineate the molecular mechanisms that may underlie this process.

#### 2.2.3 Breakage-fusion-bridge cycles

Breakage-fusion-bridge(BFB) cycle was first elucidated by Barbara McClintock in maize, and was among the classical mechanisms of ecDNA formation (30, 31). BFB cycle begins with DNA breakage followed by telomere shortening and end-to-end joining of chromosomes to form an anaphase bridge with two centromeres (32). The bridge then undergoes breakage and recombination, rendering structural alterations of chromosomes (32). As daughter cells harbor chromosomes with an aberrant number of centromeres, the cycle continues in daughter cells. End products of several rounds of BFB cycles could rearrange and form ecDNA (30, 33). After several rounds of cell cycles, the BFB cycle mechanism results in gene amplification and chromosomal arrangement, contributing to cancer progression (34).

Several studies supported that BFB cycle was involved in the formation of ecDNA. An early study indicated that in hamster cells, BFB cycle could form ecDNA, resulting in unequal segregation and amplification of the adenylate deaminase 2 (*AMPD2*) gene (35). The study also suggested that the circulation of chromosomal segments could form ecDNA (35). Other *in vitro* studies indicated that BFB was associated with chromothripsis and the development of chemotherapeutic drug resistance (36, 37). BFB cycles triggered a series of events that were related to DNA damage and chromothripsis, possibly promoting the progression and heterogeneity of tumor (37).

#### 2.2.4 Fork stalling and template switching

ecDNA may also originate from replication fork stalling and template switching, as demonstrated by breakpoint analysis of ecDNA (38). DNA replication fork stops where a DNA lesion occurs on the template strand, and the lagging strand dissociates and participates in the DNA synthesis of a neighboring replication fork. The lagging strand returns to bind the original template strand after several rounds of dissociation and invasion of the nearby fork. The returned lagging strand contains a non-complementary sequence, which bulges out as single-stranded DNA. The single-stranded DNA may then undergo replication and generates ecDNA (39).

#### 2.2.5 Combination of pre-existed ecDNA

ecDNA continues to evolve readily once it is formed. A previous study indicated that larger ecDNA could be formed by combining two smaller ecDNA, as shown in the example of ecDNA carrying KRAS in esophageal cancer (16). Analysis of WGS data of pediatric patients with glioblastoma multiforme suggested that newly formed ecDNA could be found in the relapse sample compared to the diagnosis sample (40). New ecDNA could be formed from either chromosomal DNA, or secondary alteration of existing ecDNA (40). The presence and quantity of ecDNA rely on the functions of ecDNA to promote tumor expansion and drug resistance (40).

### 2.3 Functions of ecDNA

**Figure 2:**
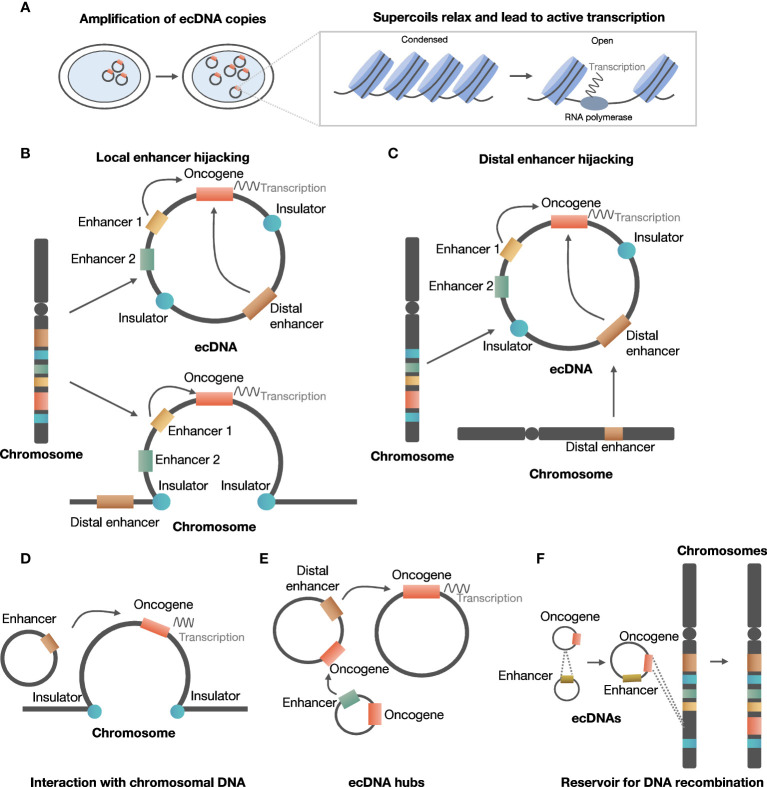
Roles of ecDNA in development and evolution of cancer. **(A)** ecDNA increases copy number of oncogene and chromatin accessibility. **(B)** ecDNA can hijack local enhancers to promote oncogene expression. **(C)** ecDNA can hijack distal chromosomal enhancers to promote oncogene expression. **(D)** ecDNA interacts with chromosomal DNA to regulate chromosomal oncogene expression. **(E)** ecDNA aggregates to form ecDNA hubs. **(F)** ecDNA serves as a reservoir for DNA recombination.

ecDNA is involved in the development and evolution of cancer in several ways, including promoting oncogene expression by increasing copy number or increasing chromosomal interaction, and serving as a reservoir for DNA recombination by reintegrating into and being excised from chromosomes (**Figure 2**).

#### 2.3.1 ecDNA enhances oncogene expression by increasing copy number and chromatin accessibility

Amplification of oncogene on ecDNA was commonly present in various types of cancer (5, 41). For example, in a study of glioblastoma cells, several oncogenes were found on ecDNA, including *MYC*, *MYCN*, *EGFR*, *PDGFRA*, *MET*, the *MECOM*–*PIK3CA*–*SOX2* gene cluster, and the *CDK4*–*MDM2* gene cluster (42). In addition, 22 of the 25 ecDNAs detected were found to carry an oncogene in multiple tumor samples from different patients (42).

ecDNA could increase copy number by focal amplification, thus increasing oncogene expression. Evidence supported that ecDNA contributed to focal amplification of oncogenes to increase copy number. A previous study suggested that oncogenes carried by ecDNA can explain the focal amplification in about half of the WGS samples from tumor tissues and cell lines derived from patients (5). Consistently, in a neuroblastoma cell line, the majority of focal amplifications (85.7%) identified by WGS coincided with ecDNA-related oncogenes (23). Haplotype phasing showed that ecDNA was exclusively derived from the amplified *MYCN* allele, strongly supporting ecDNA as an important source of focal amplification (23). Furthermore, increased copy number on ecDNA was related to elevated expression of oncogenes. For instance, analysis of allele-specific messenger RNA expression (ASE) in the same cell line confirmed that the increased gene expression was derived from ecDNA (23). Similarly, analysis of cancer cell lines and clinical samples suggested that a high copy of ecDNA was detected in cancer, and an increased copy number of ecDNA was related to increased expression of oncogenes (16).

In addition, evidence suggested that ecDNA can enhance chromatin accessibility to increase oncogene expression (40, 42). As is released from chromatin, ecDNA lacks higher-order compaction that is typical of chromosomes and displays significantly enhanced chromatin accessibility. A study utilized ATAC-seq and ATAC-see to study the chromatin accessibility of ecDNA, revealing a significantly increased number of ATAC-seq peaks compared with chromosomal DNA in all phases of the cell cycle. Combined analysis of RNA-seq results suggested that the increased accessibility contributed largely to the oncogene expression (16). Even after adjusting for copy number, oncogenes on ecDNA could achieve a higher level of expression compared to oncogenes on linear chromosomes, which might be related to increased accessibility of chromatin (6).

#### 2.3.2 ecDNA hijacks enhancers to promote oncogene expression

It has been shown that ecDNA can create ultra-long chromatin contacts that can promote oncogene expression, through local or distal enhancer hijacking.

In the local enhancer hijacking model, local upstream enhancers and oncogenes can circularize and form ecDNA, thus favoring interaction between enhancer and oncogene to facilitate expression. For example, *EGFR* and two active enhancers were co-amplified and were maintained on circular ecDNA in glioblastoma samples from TCGA (25). ChlP-Seq indicated that the formation of ecDNA could bring non-adjoining segments together, and enable interaction between upstream enhancers and *EGFR* promoters originally separated by insulators (25). Further investigation suggested that 30 of 43 amplified oncogenes in various types of tumors exhibited co-amplification with distal enhancers (25).

In the distal enhancer hijacking model, distal enhancers and oncogene on the same or other chromosome undergo circularization and form ecDNA. In neuroblastoma cell lines, *MYCN* was co-amplified with distal enhancer e4, and chromatin capture analysis indicated that e4 interacted with the promoter of *MYCN* to enhance *MYCN* expression (43).

Moreover, hijacking enhancers by forming ecDNA could modulate cell viability by regulating oncogene expression, as demonstrated in the case of *EGFR* in glioblastoma and *MYCN* in neuroblastoma (16, 43).

#### 2.3.3 ecDNA interacts with chromosomal DNA or form ecDNA hubs to regulate oncogene expression

ecDNA can interact with chromosomal DNA or form an ecDNA hub capable of regulating gene expression.

ecDNA serves as a mobile trans-acting component to enhance transcription by interacting with chromosomal DNA. In three cell lines derived from glioblastoma patients, Hi-C analysis indicated that two of the three cell lines were positive for ecDNA, and ecDNA interacted with chromosomal non-coding sequences rich in H3K27ac signals (44). Moreover, the interaction between ecDNA and chromatin was related to transcriptional activity, and artificially synthesized circular DNA mimicking ecDNA increased chromosomal oncogene expression in ecDNA-negative cells (44). Therefore, the above evidence supported that ecDNA was a potent trans-acting regulator of oncogene expression by interacting with chromosomal DNA.

ecDNAs could also regulate oncogene expression by contacting each other to form ecDNA hubs. A recent study suggested that 10 to 100 ecDNAs could form ecDNA hubs, in which the bromodomain and extra-terminal domain (BET) protein was shown to be essential for maintaining the structure of ecDNA hubs (45). MYC ecDNA lack of enhancers could receive regulation of enhancers in ecDNA hubs, suggesting that the formation of ecDNA hubs facilitated interaction between oncogenes and enhancers to promote oncogene expression (45).

#### 2.3.4 ecDNA serves as a reservoir for DNA recombination

Another important function of ecDNA is that it serves as a reservoir for DNA recombination.

One study suggested that ecDNA could form from extrachromosomal circular elements (46), and other studies have shown that ecDNA could also be generated from another ecDNA by excision or ligation of DNA fragments (47, 48).

In addition, ecDNA can reintegrate into linear chromosomes, causing intra- and inter-chromosomal rearrangements. Koche et al. found evidence that ecDNA drives oncogenic genome remodeling in neuroblastoma cells through reintegration into nuclear chromosomes, at that this phenomenon was associated with adverse clinical outcomes (23). In a glioblastoma multiforme (GBM) cell line, a high copy number of the *EGFRvIII* mutant allele is carried on ecDNA with a consistent circular structure of 1.29 Mb (49). Tandem duplications containing multiple copies of *EGFRvIII*, resulting from the reintegration of the *EGFRvIII*-containing ecDNA elements into the genome, were found in a subclone carrying *EGFRvIII* exclusively on chromosomal homogeneous staining regions (HSRs) (49). Meanwhile, the structural analysis revealed that the fine structure of the *EGFRvIII* amplicon was conserved in the subcloned and parental cells, suggesting that ecDNA dynamically relocates into chromosomal HSRs while retaining key structural features (49).

Various analyses of genomic rearrangements in cancer cells suggest that ecDNA reintegration mediates genome remodeling. First, an analysis of WGS data suggested that most intra- and inter-chromosomal rearrangements coincided with regions of extrachromosomal circularisation in neuroblastoma genomes, supporting the idea of ecDNA-mediated genome remodeling (23). Furthermore, visual inspection of Circos plots of several neuroblastoma cell lines showed that inter-chromosomal rearrangements at circularisation loci often displayed a tree-like pattern (28), which is indicative of clusters of inter-chromosomal rearrangements that originate from the same loci and extend to other distant regions of the genome (23). Thus, the association between circularisation loci and tree-shaped patterns in Circos plots supports that ecDNA can serve as a substrate for genomic rearrangements (23). In addition, analyzing phased heterozygous single-nucleotide polymorphisms (SNPs) near integration breakpoints with allele-specific PCR, the majority of rearrangement recipient sites (83.3%) were classified as circular integrations, further confirming that ecDNA is an important source of inter-chromosomal rearrangement by integration (23).

## 3 Detection methods

Mature, stable, and low-cost assays are essential for the application of ecDNA in clinical oncology. Currently, technologies based on fluorescence microscopy imaging and sequencing and their downstream algorithms have been proposed (**Table 1**).

**Table 1:** Detection tools for ecDNA.

Tool	Citation	Feature	Advantages	Disadvantages
Fluorescence microscopy-based tools				
ECdetect	(5)	Using two coarse adaptive thresholds to segment DNA and ecDNA from DAPI-stained images separately	High consistency with visual detection	Loss of small ecDNA signals due to high signal-to-noise ratio
ecSeg	(50)	Combinational detection of ecDNA and fish probe signal with U-net, a deep neural network.	Higher recall rate and F1 score than Ecdetect	Limited generalization ability and requirement of fine-tuning in each type of input data
Sequencing-based tools				
Circle-seq	(51)	Isolating ecDNA with alkaline treatment and column chromatography and then sequencing	Enrichment of ecDNA with high specificity; Full reading length for each ecDNA	Sophisticated experiment protocol and high sample size requirement
Circle_finder	(52)	Purify ecDNA with ATP–dependent exonuclease and then amplify with random primer.	Demonstrating the potential of ecDNA as a tumor diagnosis biomarker	Biased towards ecDNA with a short length
Circle-Map	(53)	Accurately detect ecDNA from short-read data crossing circle junctions	Accurately alignment of even very short soft clips (> 4 nts)	inapplicable to ecDNA with variations
ecc_finder	(54)	Identify ecDNA from Nanopore reads.	Detection of ecDNA originated from repeated loci.	Requirement of Nanopore reads.
AmpliconArchitect	(55)	Detection of amplicons on ecDNA with WGS data	Reconstruction of inexpensive short-read WGS data	The cutoff is set manually and limited accuracy.
AmpliconReconstructor	(56)	Combining NGS and OM data to reconstruct ecDNA with high fidelity.	High resolution of the breaking point detection	Extra input of the optical mapping (OM) of long DNA fragments
CRISPR-based tools				
ecTag	(57)	Using CRISPR-Cas and Casilio system to visualize ecDNA *in vivo*	Living cell imaging	gDNA design relies largely on the accurate identification of breaking point
CRISPR-CATCH	(58)	Enrich and profile genetic and epigenetic features of megabase-sized ecDNA	Characterize ecDNA at base resolution as well as the epigenetic landscape	Applicable for only large ecDNA molecule

### 3.1 Fluorescence microscopy-based tools

ecDNA can be visualized with fluorescence microscopy, with the spatial location of focal amplifications revealed. Previous studies indicate ecDNA can be stained with the fluorescent dye DAPI (4,6-diamidino-2-phenylindole) and confirmed through genomic DNA and centromeric FISH (fluorescence *in situ* hybridization) probes (5). In interphase cells, FISH probes against target oncogenes generally reveal positive sites for both chromosomal and extrachromosomal genes. While during metaphase, the compact alignment of chromosomes enables unambiguous localization of specific genes within the chromosomes or ecDNA.

However, the combination of DAPI and FISH does not allow for unbiased quantification of ecDNA copy number, which is required to evaluate the inter-cell heterogeneity. To solve this problem, Turner et al. developed an image analysis software called ECdetect (5), which provides a robust, repeatable, and accurate quantification for ecDNA from DAPI-stained metaphases in a semi-automated fashion. ECdetect applies two coarse adaptive thresholds on DAPI images to precisely detect ecDNA. The initial coarse adaptive threshold recognizes the major components in the image and smaller components are discarded. Then the recognized major components are computed to detect the chromosome regions. Then, the ecDNA search region is masked and verified manually. In the search region, the second adaptive thresholding with a smaller window size that detects components with sizes between 3 pixels and 75 pixels, the size of ecDNA. Their results prove detection accuracy with the evidence that the computed result is highly correlated with visual detection (r = 0.98) in 2,572 metaphase cells.

However, several unique features of ecDNA hinder visual detection and algorithms based on conventional computer vision approaches. ecDNA is generally irregularly shaped and small-sized. Meanwhile, a typical metaphase cell image has a high noise ratio and contains a large portion of the background signal, including nuclei, and chromosomes that need to be distinguished from ecDNA. Hence, to detect ecDNA more accurately, more sophisticated tools are required. Recently, deep neural networks, specifically convolutional neural networks, demonstrated remarkable image-processing abilities on biological datasets (59). A deep neural network-based platform called ecSeg (50) has been developed for ecDNA detection. EcSeg is developed based on U-net (60), a convolutional neural network that has been long used for biomedical image segmentation. It incorporates DAPI signals and FISH data to specifically clarify the location of oncogene amplification on both ecDNA and chromosomes. With the help of ecSeg, each pixel of the metaphase cell image is classified into one of the following classes: ecDNA, chromosome, nucleus, and cytoplasm. Then, connected pixel components of ecDNA are connected to demarcate and count one single copy of ecDNA. Last, a separate post-processing step is performed to correlate objects with ecDNA and chromosomes, using FISH probe results. The test result shows ecSeg delivers better performance than ECdetect. EcSeg achieves a mean precision and recall values of 82% at the image level, while ECdetect rarely achieved recall above 50% (3). Additionally, ecSeg achieves a higher F1 score as well (50).

### 3.2 Sequencing-based tools

With the advancement of sequencing technologies, bioinformatic analyses have provided many approaches to reconstruct ecDNA from NGS data and detect ecDNA.

Circle-seq was originally designed to detect ecDNA in yeast systems (51), and has been successfully adopted by several studies on human ecDNA (23, 61). Circular DNAs are separated by concise alkaline treatment and column chromatography, then purified from linear chromosomal DNA and mitochondria DNA by DNase digestion. Then, ecDNA is further enriched by φ29 rolling circle amplification and sequenced for mapping. Another approach to purify and amplify ecDNA is Circle_finder (52), which utilizes adenosine 5’-triphosphate (ATP)–dependent exonuclease to remove linear DNA and random primers to amplify the ecDNA. Circle_finder has a bias for ecDNA with a smaller size (200-400bp) and has been proven to detect ecDNA from the serum of cancer patients (62).

Circle-Map (53) has been developed to accurately detect circular DNA in genome sequencing data. Standard short read aligners from WGS do not reliably detect split-reads in the sequencing result that indicate circle breaking points, especially when read signals are shorter than 19 base pairs. Thus, Circle-Map utilizes discordantly mapped paired-end reads to realign the soft clipped parts resulted from breakpoints, which enables a more accurate detection of circle breakpoints. The result shows very short soft clips(>4nt) can be accurately aligned with Circle-Map.

Since ecDNA may account for focal amplification in almost half of the cancer samples (5), AmpliconArchitect (55) was developed to reconstruct ecDNA amplicon (non-overlapping amplified genomic components) structures with whole-genome sequencing data(WGS). AmpliconArchitect is an extension of structural variant (SV) analyses. In this algorithm, short, pair-end reads mapped to the reference genome and a seed interval in an amplicon are input and used to search for other intervals in the amplicon. SV analysis is then conducted to build a breakpoint graph, and copy number variant (CNV) analysis is carried out by optimizing a balanced flow on the breakpoint graph. However, the construction of a breakpoint graph can be inaccurate due to the duplicated regions inside the amplicon and leads to errors in the estimation of copy number. Thus, AmpliconReconstructor (AR) algorithm is proposed to refine the breakpoint graph reconstruction, by integrating the long-range optical mapping (OM) of long DNA fragments with NGS data. AR reconstructs genomic scaffold with OM data before the identification of breakpoint graphs, and then reconstructs ecDNA with high fidelity, which can not only provide accurate quantification of ecDNA but also contribute to the understanding of the formation mechanisms of ecDNA (56).

With the advent of third-generation long-read sequencing, characterization of the full landscape of ecDNA has become possible. A newly developed algorithm, ecc_finder (54) adopts nanopore reads to identify eccDNA. ecc_finder identifies the tandem repeat patterns as candidate loci from read alignments since circular DNA will display two or more sub-read alignments in the same direction. Then it removes the reads generated from linear genome repeats according to the reference genome, which leaves true ecDNA reads and allows for the detection of ecDNA originating from repeated loci.

### 3.3 CRISPR-based tools

CRIPSR-Cas system has been widely used in sequence-specific genome editing, since Cas can be directed to a specific sequence with the help of guide RNA. This feature allows researchers to design CRISPR-based visualization tools that target specific gene sequences carried by ecDNA and highlight ecDNA with fluorescent markers. ecTag (57), a CRISPR-based visualization pipeline, first detects ecDNA breakpoint junctions with AmpliconArchitect, and then designs sgRNA that targets the breakpoint sequence. After that, the Casilio system that combines dead Cas9 labeling and Pumilio RNA then recruits multiple fluorescent protein molecules, making the visualization possible. Since the CRISPR-Cas system and Casilio system are not lethal to cells, ecTag can be used for living cell imaging. The dynamics of ecDNA during mitosis have been observed with ecTag, supporting the theory that ecDNA is not equally distributed for two daughter cells. Another CRISPR-base tool, CRISPR-CATCH (63) is originally designed for targeted cloning of genomic sequences as long as 100 kb. The tool is then adopted to enrich megabase-sized ecDNA from cell lines or patient tissues (58). This approach does not require cut or replicate of ecDNA, preserves the topological structure and molecular features of ecDNA, and allows for detailed analyses of both the genetic sequence and epigenomic landscape.

## 4 Clinical implications of ecDNA in cancer

A previous study showed that ecDNAs were prevalent in at least 17 types of cancer and were present at higher levels in WGS samples from cancer tissues and cell lines derived from patients with cancer compared with healthy controls (5). Further investigation revealed that ecDNA profiles were correlated with different types of cancer, including glioblastoma, neuroblastoma, ovarian cancer, leukemia, oesophageal cancer, gastric cardia cancer, colorectal cancer, and lung cancer (**Table 2**).

**Table 2:** ecDNA profiles in different cancers.

Cancer type	Model	Related ecDNA gene(s)	References
Glioblastoma	Human sample	*EGFR*, *MYC*, *CDK4*, *MDM2*, *PDGFRA*	(6, 42)
Neuroblastoma	Human sample	*n-MYC*	(23, 43, 64, 65)
Ovarian cancer	Human sample	*DNMT1*	(66)
Leukaemia	Human cell line	*c-MYC*	(67)
Esophageal adenocarcinoma	Human sample	*RUNX1*	(68)
Gastric cardia adenocarcinoma	Human sample	*ERBB2*, *EGFR*	(69)
Colon cancer	Human cell line	*DHFR*	(70, 71)
Lung cancer	Human sample	*MET*, *PDZRN3*, *LGR6*	(72, 73)

### 4.1 Diagnostic biomarkers

Although tumor tissue biopsy is considered the gold standard for cancer subtyping, it provides only a snapshot of the disease and is invasive and difficult to replicate. To overcome these disadvantages, liquid biopsies are gaining favor as a new diagnostic tool to complement conventional biopsies. Well-defined biomarkers used for liquid biopsy include circulating tumor cells (CTCs) (74), circulating tumor DNA (ctDNA) (75), and extracellular vesicles (EVs) (76). Also, previous studies confirm that circular DNA can pass through cell membranes much more easily than linear DNA (77), and studies have also shown that extrachromosomal circular DNA can be released into circulation from *in situ* tumors (62, 78).

ecDNA is a valuable tool for monitoring tumor progression and predicting tumor prognosis, as it can contribute greatly to oncogene copy number variation and is associated with drug resistance. For example, it has been suggested that longer ecDNAs are enriched in tumor samples from patients with lung cancer compared with paired controls (62). Recently, next-generation sequencing (NGS) of extrachromosomal DNA from plasma samples of 6 lung adenocarcinomas (LADs) and 10 healthy controls suggested that the frequencies of nine ecDNAs were higher in cancer samples (79). In addition, four ecDNAs were specifically expressed in LADs and were promising biomarkers for early diagnosis (79). Similarly, a recent study of LAD showed that the expression profiles of several ecDNAs were significantly different between LAD and normal tissues (73). When used to discriminate patients with LAD from healthy controls, serum levels of two ecDNAs achieved higher areas under the curve (AUCs) than serum carcinoembryonic antigen (CEA) and CYFRA21-1 (cytokeratin 19 fragments), which are among the most sensitive LAD tumor biomarkers (73).

### 4.2 Prognostic biomarkers

ecDNA is associated with patient prognosis in several types of cancer, with different prognostic values for different genes. Analysis of patient samples has suggested that survival is compromised in patients with ecDNA in various cancers (6). Specifically, patients with glioblastoma have a significantly shorter relapse-free time if ecDNA is detected in primary tumor samples (42). In gastric cardia adenocarcinoma, focal amplification of *ERBB2* is correlated with a better prognosis, whereas focal amplification of *EGFR* is associated with a worse prognosis (69). Further, the survival of *ERBB2*-positive patients was lower compared with *ERBB2*-negative patients within 2 years of diagnosis, but the trend was reversed after 2 years of survival (69). As focal amplification was mainly driven by ecDNA (56), the detection of *ERBB2* and *EGFR* ecDNAs should be considered as potential prognostic biomarkers in gastric cardia adenocarcinoma. Furthermore, in high-grade serous ovarian cancer (HGSOC), ecDNA with *DNMT1* was associated with a worse prognosis in primary and metastatic lesions, suggesting that ecDNA could be a prognostic indicator for patients with HGSOC (66). Therefore, evidence from different cancer types supports that circulating ecDNA could be developed as a prognostic biomarker in various contexts.

Several mechanisms have been proposed to explain the relationship between ecDNA and prognosis. ecDNA can actively contribute to genome remodeling and lead to important functional and clinical consequences (23). In addition, ecDNA can contribute to poor outcomes through copy number variation (80). For example, focal amplification has been shown to be associated with patient mortality (80), and the importance of oncogene amplification in cancer pathogenesis is well recognized (81). Genomic amplification can result from double-strand break events such as tandem duplication (82), break–fusion–bridge cycles (83), and chromothripsis (19).

### 4.3 Drug resistance

ecDNA also contributes to resistance to targeted therapy. Oncogene amplicons carried by ecDNA can accelerate the development of intra-tumoral heterogeneity. As a result, a subpopulation of cells is more likely to express the untargeted oncogenes at levels that maximise tumor proliferation and survival, making the tumor more aggressive and resistant to previous treatments (5, 84). For example, amplification of *DHFR* ecDNA has been correlated with methotrexate resistance in *in vitro* studies (49, 85, 86), and increased copies of *DHFR* on ecDNA were observed in the methotrexate-resistant S-180 murine cell line. In addition, a stable amplification of *DHFR* was formed during the culture of S-180 cells with methotrexate (86). Similarly, ERK inhibitor-treated lung cancer or melanoma xenografts had amplification of *BRAF^V600E^*, and this study further showed that combined treatment with RAF, MEK, and ERK inhibitors could inhibit the expansion of high copy number *BRAF^V600E^* subclones (87). A recent study showed that *MET* ecDNA amplification correlated with resistance to ROS1 tyrosine kinase inhibitors both *in vitro* and *in vivo*, indicating that *MET* detection and targeting could be a strategy to overcome resistance to this class of drugs (72). In addition, amplification of *RAB3B* on ecDNA could confer cisplatin resistance by inducing autophagy in a hypopharyngeal squamous cell carcinoma (HSCC) cell line (88). Thus, increased ecDNA copy number is a common mechanism of drug resistance occurring during cancer treatment, and strategies to prevent or target amplification of oncogenes or drug resistance genes carried by ecDNAs could be used to combat adaptive responses to cancer therapies.

Studies have also shown that ecDNA can serve as a latent reservoir for genomic alterations that can be targeted for therapeutic purposes. Under changing environmental conditions, the number of amplicons in ecDNA can be naturally regulated and even reduced to zero, resulting in a complete loss of ecDNA (24, 89). Drug treatment could induce resistance by decreasing ecDNA copy number, whereas ecDNA could regain its copy number after drug withdrawal, such as has been shown for the response of EGFR ecDNA to erlotinib treatment (89). Therefore, the recovery of drug-sensitive targets on ecDNAs should also be considered as a means to overcome drug resistance.

### 4.4 Therapeutic targets

As ecDNA plays an essential role in cancer development and drug resistance, targeting ecDNA is emerging as a new approach to cancer treatment. Hydroxyurea was able to restore vinblastine sensitivity of cancer cells by reducing drug resistance genes on ecDNA in human and hamster cell lines (90). Another study showed that low doses of hydroxyurea could reduce ecDNAs in cancer cells in some patients, resulting in a prolonged period of stable disease, although the study was limited by a small sample size (91). Hydroxyurea cannot be used as a clinical anti-cancer treatment, but new drugs could be developed based on this mechanism to reduce extrachromosomal amplicons with the aim of sensitizing cancer cells to chemotherapy.

PARP-dependent DNA repair following chromothripsis can re-integrate ecDNA into the genome, thus promoting oncogene amplification and drug resistance (36). Therefore, adding PARP inhibitors to standard chemotherapy may restrain gene amplification requiring PARP-dependent end-joining DNA repair, thus suppressing tumor progression and drug resistance (36). However, targeting PARP-dependent DNA repair could also increase genome instability or generate new genetic variations that could accelerate cancer progression. DNA repair inhibitors could thus be used as adjunct therapy to existing anti-cancer drugs, although their potential side effects should be carefully considered.

Another potential therapeutic target is ecDNA interactions. Previous studies have shown that ecDNA can interact with each other to form an ecDNA hub and induce overexpression of oncogenes, and that BET is essential for these interactions (45). Therefore, it is possible that a BET inhibitor may be able to dissociate ecDNA hubs and thus improve treatment response. Further research into the formation and maintenance of ecDNA and ecDNA hubs is needed before BET inhibitors can be used in cancer treatment.

In addition, a recent study showed that ecDNA correlates with immune evasion of cancer based on WGS and gene expression data (92). In tumors with ecDNA, the number of immune cells and the expression of antigen-presenting genes were reduced, suggesting that the presence of ecDNA may contribute to immune evasion in cancer (92). Thus, it is possible that reducing ecDNA and inducing antigen presentation pathways could restore immune surveillance against cancer. Future studies to validate and elucidate the relationship between ecDNA, the immune system, and cancer could have broad implications for various therapeutic approaches, including identifying combination therapy strategies with immunotherapeutics.

**Table 3** summarizes current clinical implications of ecDNA in cancer.

**Table 3:** Clinical implications of ecDNA in cancer.

Category	Potential implications	References
Diagnostic biomarker	• ecDNA could serve as diagnostic biomarkers in plasma samples for lung cancer.	(62, 73, 79)
Prognostic biomarker	• Some ecDNA were associated with compromised survival in various cancers, e.g. glioblastoma and HGSOC. • Some ecDNA were associated with better prognosis in gastric cardia adenocarcinoma.	(6, 42)
		(56)
Drug resistance	• Oncogene amplicons carried by ecDNA can accelerate the development of intra-tumoral heterogeneity. • ecDNA can serve as a latent reservoir for genomic alterations.	(5, 49, 72, 84–88)
		(24, 89)
Therapeutic target	• Hydroxyurea restored drug sensitivity. • PARP inhibitors may target the reintegration of ecDNA. • BET inhibitors may target ecDNA hubs. • Treatments could be developed to induce antigen presentation pathways.	(90, 91)
		(36)
		(45)
		(92)

## 5 Concluding remarks and future perspectives

ecDNA has been detected in various tumor tissues, and increasing evidence suggests that it plays an essential role in tumor progression and heterogeneity. ecDNA can be generated by chromothripsis, and other mechanisms that are likely to generate ecDNA are being characterized. The genes and regulatory elements carried by ecDNA can upregulate gene expression, introduce mutations through reintegration, and increase tumor heterogeneity through non-Mendelian inheritance. ecDNA is also associated with patient prognosis and drug resistance in several cancer types, therefore detection methods for ecDNA in liquid biopsies could be developed for diagnostic and prognostic applications as well as to inform therapeutic strategies to overcome drug resistance.

Many important questions need to be addressed before ecDNA can be applied in clinical practice. To develop effective ecDNA-targeted therapy, a clearer understanding of the molecular mechanisms that underlie ecDNA dynamics and how ecDNA affects interactions between cancer cells and the immune system is required. In addition, topological configurations in ecDNA have been suggested to contribute to ecDNA function, and further studies on this topic are needed. Additional research should also be conducted to differentiate ecDNA found in normal tissues versus ecDNA detected in tumors, if possible. The half-life and abundance of ecDNA also need to be studied to determine whether ecDNA can serve as a biomarker that can be detected in liquid biopsies. Bioinformatic analyses will be useful to identify pathological features of ecDNA and to further define relationships between ecDNA molecular characteristics and cancer.

In conclusion, our understanding of ecDNA and its role in tumor progression and evolution has significantly grown in recent years, but many questions about the functions and clinical implications of ecDNA remain unresolved. As the role of ecDNA in the pathogenesis of cancer is clarified, the features of ecDNA are expected to be exploitable for the diagnosis, prognosis, and treatment of patients with cancer.

## Author contributions

YZ, LH, and YD conceived and designed the framework for the review. YD, QH, XC, and FY participate in the literature review, critical thinking and article writing. All authors contributed to the article and approved the submitted version.
